# *Chlamydia trachomatis* bacterial load, estimated by Cq values, in urogenital samples from men and women visiting the general practice, hospital or STI clinic

**DOI:** 10.1371/journal.pone.0215606

**Published:** 2019-04-19

**Authors:** Juliën N. A. P. Wijers, Christian J. P. A. Hoebe, Geneviève A. F. S. van Liere, Petra F. G. Wolffs, Nicole H. T. M. Dukers-Muijrers

**Affiliations:** 1 Department of Medical Microbiology, Care and Public Health Research Institute (CAPHRI), Maastricht University Medical Center (MUMC+), AZ Maastricht, the Netherlands; 2 Department of Sexual Health, Infectious Diseases and Environmental Health, South Limburg Public Health Service, Heerlen, the Netherlands; GGD Amsterdam, NETHERLANDS

## Abstract

**Background:**

The bacterial load of *Chlamydia trachomatis* (CT) is assumed to play a role in transmission and sequelae. We assessed urogenital CT cycle quantification (Cq) values, as an indicator for CT load, of men and women diagnosed by general practitioners (GPs), hospital physicians and the STI clinic.

**Methods:**

Urogenital CT-positive samples (n = 2,055 vaginal swabs, n = 77 cervical swabs, n = 1,519 urine samples and n = 19 urethral swabs) diagnosed by GPs, hospital physicians and the STI clinic from the Maastricht Medical Microbiology Laboratory were included (2012–2016). The outcome measure ‘urogenital Cq values’ was used as an inversely proportional measure for CT load. Among all patients, multivariate linear regression analyses were used to assess primary determinants for mean urogenital Cq values, stratified by sex. Additional clinical determinants were assessed among STI clinic patients.

**Results:**

In men, mean urogenital Cq values were similar between GPs, hospital physicians and the STI clinic (32.7 and 33.5 vs. 32.7; p>0.05). Women visiting the GP had lower urogenital Cq values than women visiting the STI clinic (30.2 vs. 30.9; p = <0.001). Women visiting the hospital had higher urogenital Cq values than women visiting the STI clinic (32.4 vs. 30.9; p = <0.001). Among STI clinic women, urogenital Cq values were lower in women with concurrent anorectal CT and in rectally untested women compared to anorectal CT-negative women (30.7 and 30.6 vs. 33.9; p = <0.001).

**Conclusion:**

Men visiting different STI care providers had similar urogenital Cq values, which could be an indicator for similar CT loads. The lower Cq values of women visiting the GP compared to women visiting the STI clinic could be an indicator for higher CT loads and likely higher transmission potential. Notably, urogenital Cq values of STI clinic women were much lower (>3 Cq) when STI clinic women also had anorectal CT. This finding could indicate higher urogenital CT loads and likely higher chances of transmission and sequelae.

## Introduction

*Chlamydia trachomatis* (CT) is the most prevalent bacterial sexual transmitted infection (STI) worldwide [1]. CT infection can increase the risk of reproductive sequelae in women, such as pelvic inflammatory disease, infertility, and ectopic pregnancy [2].

In several countries, such as Australia, the United Kingdom and the Netherlands, general practitioners (GPs) have a significant role in STI healthcare, as a large proportion of CT infections are diagnosed by GPs [3–6]. Other STI care providers include STI clinics, genitourinary medicine (GUM) clinics and hospital physicians [5, 7, 8]. STI care providers test different populations of patients. Studies have shown differences in age, sex, race and socioeconomic (SES) characteristics of patients visiting different STI care providers [7–9]. CT-positive patients visiting different STI care providers also differ in characteristics such as age, sex, race and SES [7, 8]. Potentially CT-positive patients of different STI care providers also differ in CT bacterial load (hereafter CT load).

The CT load, often expressed as the number of CT bacteria present per milliliter, has been studied over several years [10]. An earlier report by our study group showed comparable urogenital CT load in men and women participating in a Dutch population-based CT screening and STI clinic visitors, arguing similar chances of transmission and sequelae [11]. Previous studies assessing CT load included separate patient populations of STI care providers, such as the STI clinic and GPs [10, 12–14]. Nevertheless, GP, hospital and STI clinic patient populations have never before been compared regarding CT load. Comparing the CT load of patients visiting different STI care providers could expand our understanding of CT-infected patient populations served by our STI care services.

Currently, it is not known what determines a high bacterial CT load in a patient and what its consequences are. Symptoms might be associated with a higher CT load but this remains a matter of debate [10]. It could be relevant to assess determinants for high CT loads. For example, in viral STIs, such as herpes simplex virus and human immunodeficiency virus (HIV), it has been shown that higher viral loads increase transmission potential [10]. However, it is unknown whether this also applies to CT load [10].

Concurrent urogenital and anorectal infections are common among women visiting the STI clinic, i.e. more than 70% of women with urogenital infections also have an anorectal infection [15, 16]. Current guidelines advocate anorectal testing in women based on indication, i.e. after self-report of anal sex and/or symptoms [15]. However, as GPs rarely test women anorectally it remains unknown whether anorectal infections are common among women visiting the GP [17].

Our main objective was to compare the urogenital CT Cq values, as an indicator for CT load, between CT-positive patients tested by GPs, hospital physicians and the STI clinic to obtain more insight in the CT loads of different populations. Our second objective was to assess which clinical determinants were associated with urogenital Cq values.

## Methods and materials

### Ethics statement

The medical ethics committee of the Maastricht University Medical Center (Maastricht, the Netherlands) approved this study (METC 2017–0251) and waived the need for consent to be collected from participants. Since retrospective data originated from regular care and were analyzed anonymously, no further informed consent for data analysis was obtained.

### Study population

In this cross-sectional study, data from 3,899 test consultations of urogenital CT-positive patients ≥16 years (from n = 38,599 consultations; 10.1% CT positive) were obtained from the Medical Microbiology Laboratory of Maastricht University Medical Center (MUMC+) from January 2012 through May 2016. Data included consultations performed by GPs, hospital physicians and the STI clinic. The majority of hospital consultations were performed by gynecologists (42,5%; n = 48) followed by internists (22.1%; n = 25). The proportions of urogenital CT tests performed by the GP, hospital physicians and STI clinic do not reflect the real distribution of STI care provider testing in our region, as we included different geographic serving areas for the STI care providers.

The laboratory provides the same instruction methods for collecting CT samples for GPs, hospital physicians and STI clinic using the same sampling materials. The CT samples were daily transported to the laboratory. All samples were collected in a standardized way and were analyzed in the same laboratory.

All samples were tested for CT with a nucleic acid amplification test (NAAT) (COBAS 4800, Roche Diagnostics, Basel, Switzerland), as per the manufacturer’s protocol [11]. The NAAT-derived cycle quantification (Cq) value is commonly used as a proxy for bacterial load in other infectious diseases; a low Cq value indicates a high load, and vice versa [18–20].

**Fig 1:**
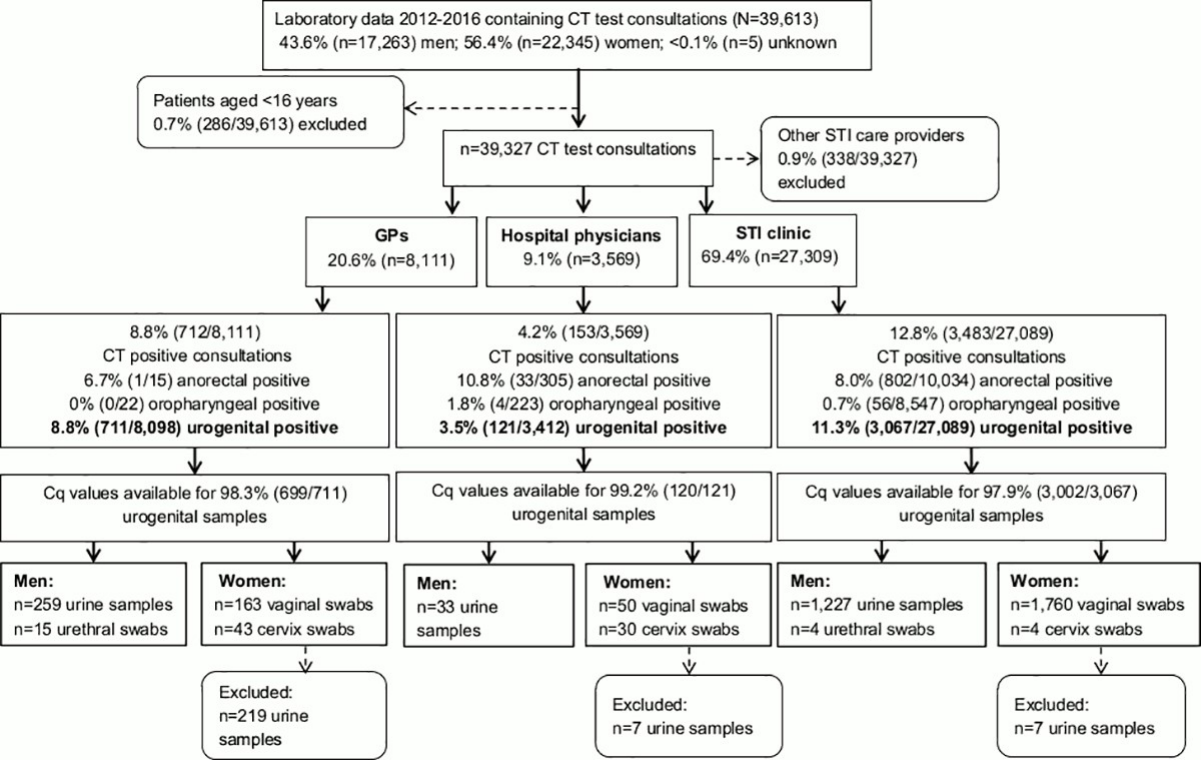
Flowchart, including *Chlamydia trachomatis* samples taken by general practitioners, hospital physicians and the STI clinic between January 2012 and May 2016.

The study population included data from all samples for which the Cq value could be retrieved (98.0%; 3,821/3,899) (Fig 1). For men, data from urine and urethral swabs were available. Mean Cq values from urine (n = 1,519, M = 32.69, SD = 3.20) and urethral swabs (n = 19, M = 32.88, SD = 4.80) were comparable in men (p = 0.86). These data were merged together as ‘urogenital Cq values’.

For women, data from urine, vaginal swabs and cervix swabs were available. Mean Cq values from vaginal swabs (n = 1,973, M = 30.86, SD = 3.73) and cervix swabs (n = 77, M = 30.58, SD = 4.24) were comparable in women (p = 0.51). The data were merged together as ‘urogenital Cq values’. Mean Cq values from vaginal swabs (n = 1,973, M = 30.86, SD = 3.73) and urine (n = 233, M = 33.28, SD = 3.60) were significantly different (p = <0.001). Data from urine samples of women were excluded (n = 233), as international guidelines advocate vaginal swabs, as the diagnostic value (sensitivity and specificity) for detecting CT in urine samples is lower compared to vaginal swabs [21–24]. The majority of urine samples from women were taken by GPs (94%; Fig 1).

### Cycle quantification validation for CT load

We tested the use of the Cq value as a proxy for CT load by comparing derived Cq values with quantified CT load values from our previous studies [11, 25, 26]. CT load values were available from a subset of the STI clinic population, i.e. n = 103 vaginal swabs from women (S1 Table). In short, we quantified CT load by an in-house TaqMan real-time qPCR to quantify CT *OmpA*-gene copies/ml [11]. A full description of the CT load quantification has been described elsewhere [11].

S1 Fig shows the high correlation between vaginal Cq values and vaginal CT load (CT/ml log10) (Pearson’s r: -0.80, n = 103, p = <0.001). Therefore, the Cq value is a valid inversely proportional proxy for CT load.

### Statistical analyses

The main objective was to compare the outcome measure, i.e. ‘urogenital Cq values’, between the populations visiting different STI care providers. Therefore, the main determinant was STI care provider (GP, hospital physician, or STI clinic).

Analyses were stratified for men and women since CT load varies by sample type and sex [10]. Baseline characteristics were compared between the GP, hospital physician and the STI clinic CT-positive populations using chi-square tests. Univariable and multivariable linear regression analyses were performed to test the association between the main determinant and the outcome, controlling for putative confounders. The putative confounders were available for the whole study population and included age in years (<25, ≥25), SES (low, medium, high, unknown), *Neisseria gonorrhoeae* (NG) urogenital positive (yes, no, not tested) and HIV positivity (yes, no, not tested). Dutch SES scores based on income, education level and employment were extracted from the Netherlands Institute for Social Research (http://www.scp.nl) per four-digit postal code area of the patient.

Our second objective was to assess potential associations between the outcome measure and clinical determinants (available for the STI clinic population), including concurrent anorectal CT infection (no anorectal test, yes, no), urogenital symptoms (unknown, yes, no), oropharyngeal symptoms (unknown, yes, no), proctitis (unknown, yes, no) and, for men, sexual preference (unknown, MSM, heterosexual men).

**Fig 2:**
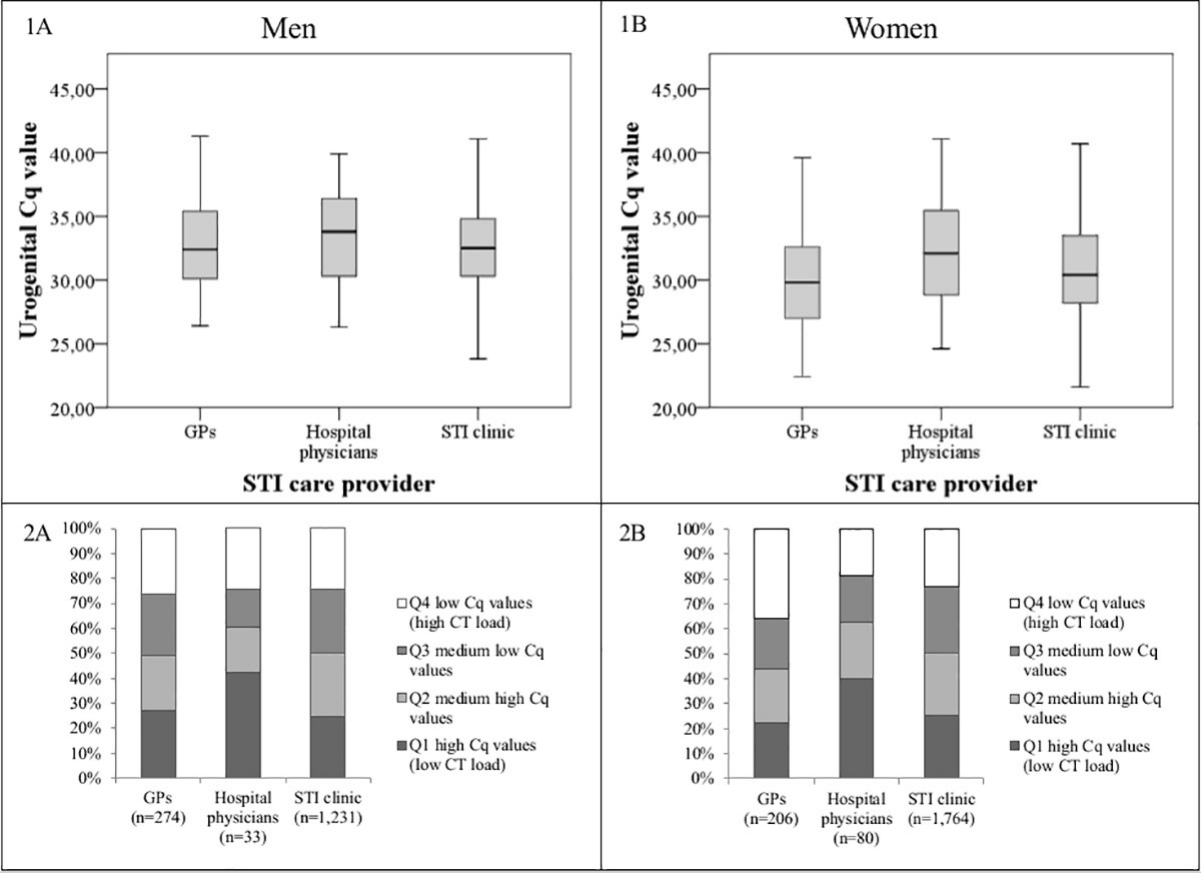
Boxplots and bar diagrams showing the distribution of high- and low Cq values for the GP, hospital and STI clinic population. (1) Boxplots showing the distribution of urogenital Cq values between the GP, hospital physicians and STI clinic population for men (A) and women (B). (2) Bar diagrams showing the frequencies of low (high CT load), medium low, medium high and high (low CT load) urogenital Cq values based on quartiles per STI provider for men (A) and women (B).

For all linear regression analyses, determinants with p<0.05 in the univariable model were included in the multivariable model. To test our main objective, the main determinant ‘STI care provider’ was entered in the multivariable model. Means, betas and 95% confidence intervals (CI) were calculated. Finally, the proportions of low and high Cq values were assessed between the STI care providers based on quartiles and are depicted in Fig 2. Analyses were performed using SPSS V21 (IBM SPSS Statistics for Windows, IBM Corporation, Armonk, New York, USA). A p value of *<*0.05 was considered statistically significant.

## Results

### Study population

Of the 3,588 urogenital samples in the dataset, 42.9% (n = 1,538) were samples from men, and 57.1% (n = 2,050) were samples from women. Baseline characteristics (age, SES, NG and HIV) differed between the CT-positive populations visiting GPs, hospital physicians and the STI clinic in both men and women (Table 1).

**Table 1:** Baseline characteristics of urogenital *Chlamydia trachomatis*-positive patient populations visiting the general practitioner, hospital physician or STI clinic for men and women separately, 2012–2016.

	Men				Women			
	GP (n = 274)	Hospital physicians (n = 33)	STI clinic^a^ (n = 1,231)	P-value	GP (n = 206)	Hospital physicians (n = 80)	STI clinic^a^ (1,764)	P-value
**Age in years, % (n)**				**<0.001**				**<0.001**
<25	37.2 (102)	15.2 (5)	58.1 (715)		52.9 (109)	55.0 (44)	81.6 (1,440)	
≥25	62.8 (172)	84.8 (28)	41.9 (516)		47.1 (97)	45.0 (36)	18.4 (324)	
**SES, % (n)**				**<0.001**				**<0.001**
Low	32.8 (90)	24.2 (8)	27.1 (333)		35.4 (73)	40.0 (32)	27.1 (478)	
Medium	32.1 (88)	33.3 (11)	28.2 (347)		30.1 (62)	30.0 (24)	25.3(446)	
High	32.1 (88)	39.4 (13)	30.5 (376)		32.0 (66)	27.5 (22)	35.2 (621)	
Unknown	2.9 (8)	3.0 (1)	14.2 (175)		2.4 (5)	2.5 (2)	12.4 (219)	
**NG urogenital positive**				**<0.001**				**<0.001**
Yes	4.0 (11)	21.2 (7)	19.5 (240)		3.9 (8)	2.5 (2)	9.3 (164)	
No	84.3 (231)	78.8 (26)	80.5 (991)		77.2(159)	82.5 (66)	90.7 (1,600)	
Not tested	11.7 (32)	0.0 (0)	0.0 (0)		18.9 (39)	15.0 (12)	0.0 (0)	
**HIV positive**				**<0.001**				**<0.001**
Yes	1.5 (4)	51.5 (17)	4.2 (52)		1.5 (3)	6.3 (5)	0.2 (4)	
No	49.6 (136)	21.2 (7)	67.3 (828)		10.2 (21)	5.0 (4)	54.8 (966)	
Not tested	48.9 (134)	27.3 (9)	28.5 (351)		88.3 (182)	88.8 (71)	45.0 (794)	

^a^ The STI clinic comprised a larger geographic area. Therefore, the data are not applicable for comparing the proportions of CT testing between STI care providers.

Abbreviations: GP, general practitioner; STI, sexually transmitted infection; SES, socioeconomic status; NG, *Neisseria gonorrhoeae*; HIV, human immunodeficiency virus.

### Urogenital Cq values of CT patients compared between STI care providers

In multivariable analyses, mean urogenital Cq values were similar in men diagnosed by GPs (M = 32.7, SD 3.2), hospital physicians (M = 33.5, SD = 3.5) and the STI clinic (M = 32.7, SD = 3.2) (p = 0.36) (Fig 2 and Table 2). Based on quartiles, the proportions of low urogenital Cq values were similar among men visiting GPs (26.3%), hospital physicians (24.2%) and the STI clinic (24.2%) (Fig 2).

**Table 2:** Primary analyses, including determinants associated with urogenital *Chlamydia trachomatis* cycle quantification threshold values for men and women.

	Men				Women			
	% (n)	Mean Cq value (SD)	B (95% CI)	Adj. B (95% CI)	% (n)	Mean Cq value (SD)	B (95% CI)	Adj. B (95% CI)
Overall	100 (1,538)	32.7 (3.2)			100 (2,050)	31.1 (3.8)		
**STI care provider**								
GP	17.8 (274)	32.7 (3.2)	0.09 (-0.34–0.51)	0.22 (-0.26–0.70)	10.0 (206)	30.2 (4.2)	**-0.68 (-1.21–-0.14)**	**-0.95 (-1.61–-0.29)**
Hospital physicians	2.1 (33)	33.5 (3.5)	0.80 (-0.32–1.91)	0.38 (-0.80–1.56)	3.9 (80)	32.4 (4.3)	**1.42 (0.58–2.26)**	**1.08 (0.15–2.01)**
STI clinic	80.0 (1,231)	32.7 (3.2)	Ref	Ref	86.0 (1,764)	30.9 (3.7)	Ref	Ref
**Age in years**								
<25	53.4 (822)	32.5 (3.1)	**-0.51 (-0.83– -0.19)**	**-0.37(-0.70–0.03)**	77.7 (1,593)	30.6 (3.7)	**-1.10 (-1.49 –-0.72)**	**-1.17 (-1.57– -0.77)**
≥25	46.6 (716)	33.0 (3.)	Ref	Ref	22.3 (457)	31.7 (3.9)	Ref	Ref
**SES**								
Low	28.0 (431)	32.9 (3.3)	Ref		28.4 (583)	31.0 (3.8)	Ref	
Medium	29.0 (446)	32.7 (3.2)	-0.15 (-0.58–0.28)		26.0 (532)	30.9 (3.7)	-0.10 (-0.54–0.34)	
High	31.0 (477)	32.7 (3.1)	-0.52 (-1.07–0.04)		34.6 (709)	30.6 (3.7)	**-0.44 (-0.85–-0.30)**	
Unknown	12.0 (184)	32.3 (3.3)	-0.33 (-0.87–0.22)		11.0 (226)	31.1 (3.7)	0.12 (-0.46–0.70)	
**NG urogenital positive**^a^								
Yes	3.8 (58)	33.9 (3.2)	Ref	Ref	2.2 (45)	31.8 (3.1)	Ref	
No	94.0 (1,446)	32.6 (3.2)	**-1.27 (-2.11–-0.42)**	**-1.20 (-2.05–-0.36)**	95.3 (1,954)	30.8 (3.7)	-0.97 (-2.08–0.14)	
Not tested	2.2 (34)	33.1 (3.7)	-0.83 (-2.19–0.53)	-0.65(-2.07–0.77)	2.5 (51)	30.9 (4.5)	-0.90 (-2.40–0.60)	
**HIV positive**								
Yes	4.7 (73)	33.8 (3.2)	Ref	Ref	0.6 (12)	33.5 (4.4)	Ref	Ref
No	79.6 (1,224)	32.7 (3.2)	**-1.13 (-1.89–-0.38)**	**-0.82 (-1.64–-0.01)**	74.6 (1,530)	30.9 (37)	**-2.57 (-4.69–-0.44)**	-1.91 (-4.06–0.25)
Not tested	15.7 (241)	32.2 (3.3)	**-1.60 (-2.44–-0.76)**	**-1.44(-2.36– -0.52)**	24.8 (508)	30.7 (3.8)	**-2.80 (-4.94–-0.65)**	-2.02 (-4.16–0.12)

^a^ For men, only GP patients were not tested for NG. For women, GP and hospital physician patients were not tested for NG.

Statistically significant associations are depicted in bold (p<0.05). Abbreviations: Cq, cycle quantification threshold; GP, general practitioner; STI, sexually transmitted infection; SES, socioeconomic status; NG, *Neisseria gonorrhoeae*; HIV, human immunodeficiency virus; B, beta; CI, confidence interval.

In multivariable analyses, urogenital Cq values were lower for women visiting the GP compared to women visiting the STI clinic (M = 30.2, SD = 4.2 vs. M = 30.9, SD = 3.7, p<0.001). Urogenital Cq values were higher for women visiting hospital physicians (M = 32.4, SD = 4.3, p<0.001) compared to women visiting the STI clinic. Based on quartiles, the proportion of low urogenital Cq values of CT positive women was higher for GPs (35.9%) compared to hospital physicians (18.8%) and the STI clinic (23.2%) (Fig 2).

### Other determinants associated with urogenital Cq values

In multivariable analyses, age <25 years (compared to age ≥ 25 years), no concurrent NG (compared to concurrent NG), HIV negative and not being tested for HIV (compared to HIV positive) were associated with lower Cq values in men (Table 2).

Furthermore, age <25 years (compared to age ≥ 25 years) was associated with lower Cq values in women (Table 2).

### Clinical determinants in STI clinic patients

In multivariable analyses, having no concurrent NG (compared to NG positive), not being tested for HIV (compared to HIV positive) and having urogenital symptoms (compared to having no urogenital symptoms) were associated with lower urogenital Cq values in men (Table 3).

**Table 3:** Additional analyses, including determinants associated with urogenital *Chlamydia trachomatis* cycle threshold values for men and women visiting the STI clinic.

	Men				Women			
	% (n)	Mean Cq value (SD)	B (95% CI)	Adj. B (95% CI)	% (n)	Mean Cq value	B (95% CI)	Adj. B (95% CI)
Overall	100 (1,231)	32.7 (3.2)			100 (1,764)	30.9 (3.7)		
**Age in years**								
<25	58.1 (715)	32.4 (3.1)	**-0.53 (-0.89– -0.16)**	-0.21 (-0.58–0.17)	81.6 (1,440)	30.7 (3.6)	**-1.16 (-1.59 –-0.72)**	**-0.83 (-1.28– -0.39)**
≥25	41.9 (516)	33.0 (3.3)	Ref	Ref	18.4 (324)	31.8 (3.8)	Ref	Ref
**SES**								
Low	27.1 (333)	32.8 (3.2)	Ref		27.1 (478)	31.0 (3.7)	Ref	
Medium	28.2 (347)	32.8 (3.2)	-0.05 (-0.53–0.44)		25.3 (446)	30.9 (3.7)	-0.07 (-0.54–0.40)	
High	30.5 (376)	32.6 (3.1)	-0.19 (-0.67–0.28)		35.2 (621)	30.6 (3.6)	-0.33 (-0.77–0.10)	
Unknown	14.2 (175)	32.3 (3.3)	-0.48 (-1.07–0.11)		12.4 (219)	31.2 (3.8)	0.21 (-0.37–0.80)	
**NG urogenital positive**								
Yes	3.8 (47)	34.2 (3.2)	Ref	Ref	2.2 (38)	31.6 (3.1)	Ref	
No	96.2 (1,184)	32.6 (3.2)	**-1.59 (-2.52–-0.65)**	**-1.57 (-2.50–-0.64)**	97.8 (1,726)	30.8 (3.7)	-1.01 (-2.18–0.17)	
**HIV positive**								
Yes	4.2 (52)	34.1 (3.3)	Ref	Ref	0.2 (4)	34.0 (4.9)	Ref	
No	87.8 (1,081)	32.7 (3.2)	**-1.42 (-2.31–-0.53)**	-0.93 (-1.93–0.06)	85.3 (1,505)	30.9 (3.7)	-3.09 (-6.67–0.50)	
Not tested	8.0 (98)	31.9 (3.0)	**-2.23 (-3.30–-1.15)**	**-1.84 (-3.02–-0.65)**	14.5 (255)	30.7 (3.2)	-3.31 (-6.92–0.29)	
**Concurrent urogenital and anorectal CT infection**								
No anorectal test	83.7 (1,030)	32.5 (3.1)	-1.04 (-2.38–0.30)		73.9 (1,304)	30.6 (3.6)	**-3.28 (-3.96– -2.59)**	**-3.01(-3.71– -2.31)**
yes	4.8 (59)	33.7 (3.6)	0.09 (-0.88–1.06)		19.7 (348)	30.7 (3.3)	**-3.20 (-3.96– -2.44)**	**-3.08 (-3.84– -2.32)**
No	11.5 (142)	33.6 (3.5)	Ref		6.3 (112)	33.9 (3.6)	Ref	Ref
**Urogenital symptoms**								
Unknown	9.7 (120)	33.7 (3.3)	0.54 (-0.09–1.18)	0.39 (-0.36–1.14)	19.6 (346)	30.7 (3.5)	-0.49 (-1.00–0.02)	
Yes	50.8(625)	32.1 (3.2)	**-1.05 (-1.43– -0.68)**	**-1.11 (-1.49– -0.73)**	54.6 (963)	30.8 (3.7)	-0.43 (-0.84–-0.02)	
No	39.5 (486)	33.1 (3.1)	Ref	Ref	25.8 (455)	31.2 (3.6)	Ref	
**Oropharyngeal symptoms**								
Unknown	9.7 (120)	33.7 (3.3)	**1.10 (0.49–1.70)**	0.39 (-0.36–1.14)	19.6 (346)	30.7 (3.5)	-0.18 (-0.61–0.26	
Yes	6.0 (74)	32.0 (3.3)	-0.61 (-1.37–0.14)	-0.37 (-1.11–0.37)	7.3 (129)	31.2 (3.9)	0.28 (-0.38–0.94)	
No	84.2 (1,037)	32.6 (3.2)	Ref	Ref	73.1 (1,289)	30.9 (3.7)	Ref	
**Proctitis**								
Unknown	9.7 (120)	33.7 (3.3)	**1.15 (0.55–1.76)**	0.39 (-0.36–1.14)	19.6 (346)	30.7 (3.5)	-0.17 (-0.61–0.26)	
Yes	6.5 (80)	32.7 (3.4)	0.17 (-0.56–0.90)	0.13 (-0.60–0.87)	5.3 (93)	31.3 (4.1)	0.43 (-0.34–1.20)	
No	83.8 (1,031)	32.5 (3.2)	Ref	Ref	75.1 (1,325)	30.9 (3.7)	Ref	
**Sexual orientation**^a^								
MSM	11.9 (147)	33.5 (3.5)	Ref	Ref	na	na	na	na
Heterosexual men	72.1 (888)	32.4 (3.1)	**-1.17 (-1.73–-0.62)**	-0.53 (-1.15–0.08)	na	na	na	na
Unknown	15.9 (196)	33.3 (3.1)	-0.30 (-0.98–0.39)	-0.18 (-0.97–0.61)	na	na	na	na

^a^ Determinant only assessed among STI clinic men.

Statistically significant associations are depicted in bold (p<0.05). Abbreviations: Cq, cycle quantification threshold; GP, general practitioner; STI, sexual transmitted infection; SES, socioeconomic status; NG, *Neisseria gonorrhoeae*; HIV, human immunodeficiency virus; B, beta; CI, confidence interval; na, not applicable.

Furthermore, age <25 years (compared to age ≥ 25 years), having no anorectal CT test and having a concurrent anorectal CT infection (compared to no anorectal CT infection) were associated with lower Cq values in women (Table 3).

## Discussion

To date, our study is the first to compare urogenital Cq values, as a potential indicator for CT load, of men and women diagnosed by GPs, hospital physicians and the STI clinic. Among all STI care providers, men visiting GPs, hospital physicians and the STI clinic had similar urogenital Cq values and likely similar urogenital CT loads. Women diagnosed by GPs had lower Cq values than women visiting the STI clinic, which could be indicative for higher urogenital CT loads. Whereas women visiting the hospital had higher urogenital Cq values than women visiting the STI clinic; likely indicative for lower CT loads. Of all determinants studied, only a few determinants were statistically associated with urogenital Cq values. However, one determinant, assessed among STI clinic visitors, was notable as the adjusted mean difference was much higher (> 3 Cq) compared to all other determinants.

A strength of the current study is the large number of included samples from different STI care providers. Furthermore, the additional analyses on clinical determinants in the STI clinic population allowed us to increase our understanding of what type of patients have lower urogenital Cq values and, therefore, likely higher urogenital CT loads. Furthermore, vaginal Cq values and vaginal CT loads were highly correlated. Therefore, vaginal Cq values were a valid indicator for vaginal CT loads.

A general limitation of CT load-based studies is that the variability of low and high CT loads, and therefore high and low Cq values, in populations and individuals seems dependent of different factors, including time of diagnosis since infection; this hampers interpretation. For example, our study group assessed the natural course of the CT load during infection between screening and treatment and observed a decrease in CT load in 17–41% of the STI clinic patients dependent on sample type [26]. A limitation of the current study was that we were unable to validate Cq values for (1) other populations than the STI clinic population and (2) for urine samples of men. Therefore, it remains unclear whether Cq values of the GP- and hospital population are a valid proxy for CT load in GP- and hospital populations and whether Cq values are a valid indicator for CT loads in urine samples. Our outcome measure ‘urogenital Cq values’ provides an indication of CT load. However, estimating the number of gene copy numbers per milliliter (often expressed as CT/milliliter) would have led to more accurate estimates of CT load. The Cq values in the current study are not reproducible for all NAAT used for CT detection. For example, if our study was repeated with a Siemens Versant NAAT, different Cq values could be produced. However, the relative differences between the Cq values of, for example, the GP, hospital and STI clinic population will be the same. Furthermore, we were unable to assess whether symptoms (urogenital symptoms, oropharyngeal symptoms and proctitis) were a result of a CT infection. Therefore, the symptoms could be caused by other STI’s such as herpes simplex virus, *Mycoplasma genitalium or Trichomonas vaginalis*. However, as those STI’s occur to a much lesser extent among STI clinic visitors in the Netherlands the influence seems to be low [27, 28]. Furthermore, as we used urogenital Cq values as a proxy for CT load caution is needed when comparing our results to studies that used the actual CT load as an outcome measure.

The purpose of the current study was to provide insight in the CT load of populations visiting different STI care providers. The clinical relevance of our main finding, suggesting different CT loads between women visiting the GP, hospital and STI clinic, remains debatable as the exact role of CT load remains unknown [10]. Therefore, results of the current study will not lead to clinical consequences. Earlier, our study group deemed a difference of 1 log load (3.3 Cq) as clinically relevant to overcome potential technical variations when measuring the CT load within the same patient over time [26]. However, in the current study we averaged Cq values over an entire population, i.e. GP, hospital and STI clinic population. Therefore, even a smaller difference than 3.3 Cq could be clinically or microbiologically relevant. Still, the exact cut-off value for a relevant difference in CT load between populations remains unknown.

One determinant showed an adjusted difference of 3.08 Cq, related to anorectal CT, which was much higher than all other determinants. Dubbink and colleagues also observed a higher urogenital CT load among South-African women concurrently infected with anorectal CT [29]. It is likely that the majority of STI clinic women who were not anorectally tested also had an anorectal infection since more than 70% of the STI clinic women with a urogenital infection also have a concurrent anorectal infection [15, 16]. Indeed, urogenital Cq values were comparable for STI clinic women who had diagnosed concurrent urogenital and anorectal CT infections and STI clinic women who were not anorectally tested. Those STI clinic women who were only urogenitally CT positive and not anorectally tested were likely treated with azithromycin, as azithromycin is the first choice treatment for urogenital CT in the Netherlands [30]. Therefore, possible undiagnosed concurrent anorectal CT infections could be not adequately treated, as the efficacy of azithromycin, compared to doxycycline, may be lower for anorectal CT infections [23, 31]. Likely women with concurrent urogenital and anorectal CT infections could have higher transmission potential, yet this remains unclear. Recently, our study groups showed a borderline significant association (P = 0.054) between lower vaginal Cq values, i.e. likely higher CT loads, and not reaching microbiological cure for vaginal CT in women treated with azithromycin [32]. Therefore, some women with concurrent vaginal and (untested) anorectal infections in our study may not be microbiologically cured when treated with azithromycin as they tend to have lower Cq values and therefore potentially higher CT loads [32]. Notably, anorectal testing was rarely performed by GPs and hospital physicians (Fig 1), as has also been shown before [8, 17, 33]. However, according to international guidelines, anorectal testing should at least be performed in men and women reporting anorectal intercourse or symptoms [23, 24]. Still, it remains unknown to which extent women visiting the GP report anal intercourse. However, it is unlikely that this would be 0.2%, as a population based study showed that 10.5% of women report to have anorectal intercourse in the past year [34].

Two explanations could possibly explain the lower Cq values of women visiting the GP compared to women visiting the STI clinic. First, lower urogenital Cq values of women visiting the GP could be related with symptoms, as symptoms could be associated with higher CT loads [10]. A study by van Bergen and colleagues showed that only 20% of the patients with STI related symptoms visit the STI clinic, whereas the majority of symptomatic patients visit the GP (63%) [35]. Second, women visiting the STI clinic could have higher Cq values, and therefore likely lower CT loads, due to frequent CT infections. It has been shown that the CT load is lower when having repeat CT infections [36]. Moreover, retesting rates are higher for the STI clinic population compared to the GP population what could strengthen this explanation [37]. The higher Cq values of women visiting the hospital may be due to different sampling moments during the infection [11, 38]. It is likely that women visit the hospital at a later stageof the infection than women visiting the STI clinic, which could have impact on the Cq values, and therefore likely CT loads, as CT loads tend to decrease over time [26].

Younger women (<25 years) had significantly lower urogenital Cq values, i.e. potentially higher CT loads, than older (≥25 years) women. Others report that partial immunity acquired to past CT infection could possibly lead to lower CT load with increasing age [39]. The lower urogenital Cq values of men without concurrent NG and men who were not tested for HIV remains unexplained. Men with urogenital symptoms had lower Cq values, i.e. likely higher urogenital CT loads, than men without urogenital symptoms. Symptoms associated with higher urogenital CT load in men have been observed in several studies [11, 40, 41]. An explanation for higher urogenital CT loads could be that higher CT loads induce a greater inflammatory response [11].

We excluded all urine samples of women (n = 233). Almost all urine samples were taken by the GP (94%). GPs should consider collecting self-sampled vaginal swabs of women, as the sensitivity and specificity of vaginal swabs for detecting CT are much higher compared to urine samples [22–24].

## Conclusions

The patient characteristics of male CT patients diagnosed by GPs, hospital physicians and STI clinic differed by type of STI care provider. However, the urogenital Cq values of men were similar, arguing similar CT loads and chances of transmission and sequelae. Women visiting the GP had lower urogenital Cq values than women visiting the STI clinic, which could be an indicator for higher urogenital CT loads. Whereas women visiting the hospital had higher urogenital Cq values than women visiting the STI clinic, which could be an indicator for lower urogenital CT loads. The impact, in terms of transmission and sequelae, of lower Cq values, and likely higher CT loads, needs to be explored further. Notably, much lower urogenital Cq values, and likely higher urogenital CT loads, were observed when STI clinic women also had anorectal CT and in STI clinic women who were not anorectally tested but who are prone to have an undiagnosed anorectal infection. Likely those STI clinic women with anorectal infections have higher chances of transmission and sequelae.

## Supporting information

S1 Table Comparison of women for whom CT load values (CT/ml) were unavailable and the subset for which the CT load values were available. Abbreviations: SES, socioeconomic status; CT, *Chlamydia trachomatis*; NG, *Neisseria gonorrhoeae*; HIV, human immunodeficiency virus.
(DOCX)

S1 Fig Scatterplot showing the correlation between CT Cq values (y-axis) and CT load (CT/ml log10) (x-axis) for vaginal swabs of women (n = 103). (TIF)
