# Supplementary material for: Chlamydia trachomatis bacterial load, estimated by Cq values, in urogenital samples from men and women visiting the general practice, hospital or STI clinic
Source: PLoS One. 2019 Apr 19;14(4):e0215606. doi: 10.1371/journal.pone.0215606 (PMC6474615; doi:10.1371/journal.pone.0215606)
Supplement: S1 Table — Abbreviations: SES, socioeconomic status; CT, Chlamydia trachomatis; NG, Neisseria gonorrhoeae; HIV, human immunodeficiency virus. (DOCX) [file pone.0215606.s001.docx]

|  | Population  % (n) | Subset  % (n) | P-value |
| --- | --- | --- | --- |
| Overall | 95.0 (1,947) | 5.0 (103) |  |
| **Age** |  |  | 0.14 |
| < 25 years | 78.0 (1,519) | 71.8 (74) |  |
| ≥ 25 years | 22.0 (428) | 28.2 (29) |  |
| **SES** |  |  |  |
| Low | 28.0 (546) | 35.9 (37) | **0.04** |
| Medium | 25.6 (499) | 32.0 (33) |  |
| High | 35.0 (682) | 26.2 (27) |  |
| Unknown | 11.3 (220) | 5.8 (6) |  |
| **Concurrent anorectal CT infection** |  |  | **<0.001** |
| Yes | 12.9 (251) | 94.2 (97) |  |
| No | 5.8 (112) | 1.9 (2) |  |
| Not anorectally tested | 81.4 (1,584) | 3.9 (4) |  |
| **NG positive** |  |  | 0.17 |
| Yes | 2.3 (44) | 1.0 (1) |  |
| No | 95.1 (1,852) | 99.0 (102) |  |
| Not tested | 2.6 (51) | 0.0 (0) |  |
| **HIV** |  |  | **<0.01** |
| Positive | 0.6 (11) | 1.0 (1) |  |
| Negative | 47.5 (924) | 65.0 (67) |  |
| Not tested | 52.0 (1,012) | 34.0 (35) |  |
