# Supplementary figures and images for: Chlamydia trachomatis bacterial load, estimated by Cq values, in urogenital samples from men and women visiting the general practice, hospital or STI clinic
Source: PLoS One. 2019 Apr 19;14(4):e0215606. doi: 10.1371/journal.pone.0215606 (PMC6474615; doi:10.1371/journal.pone.0215606)

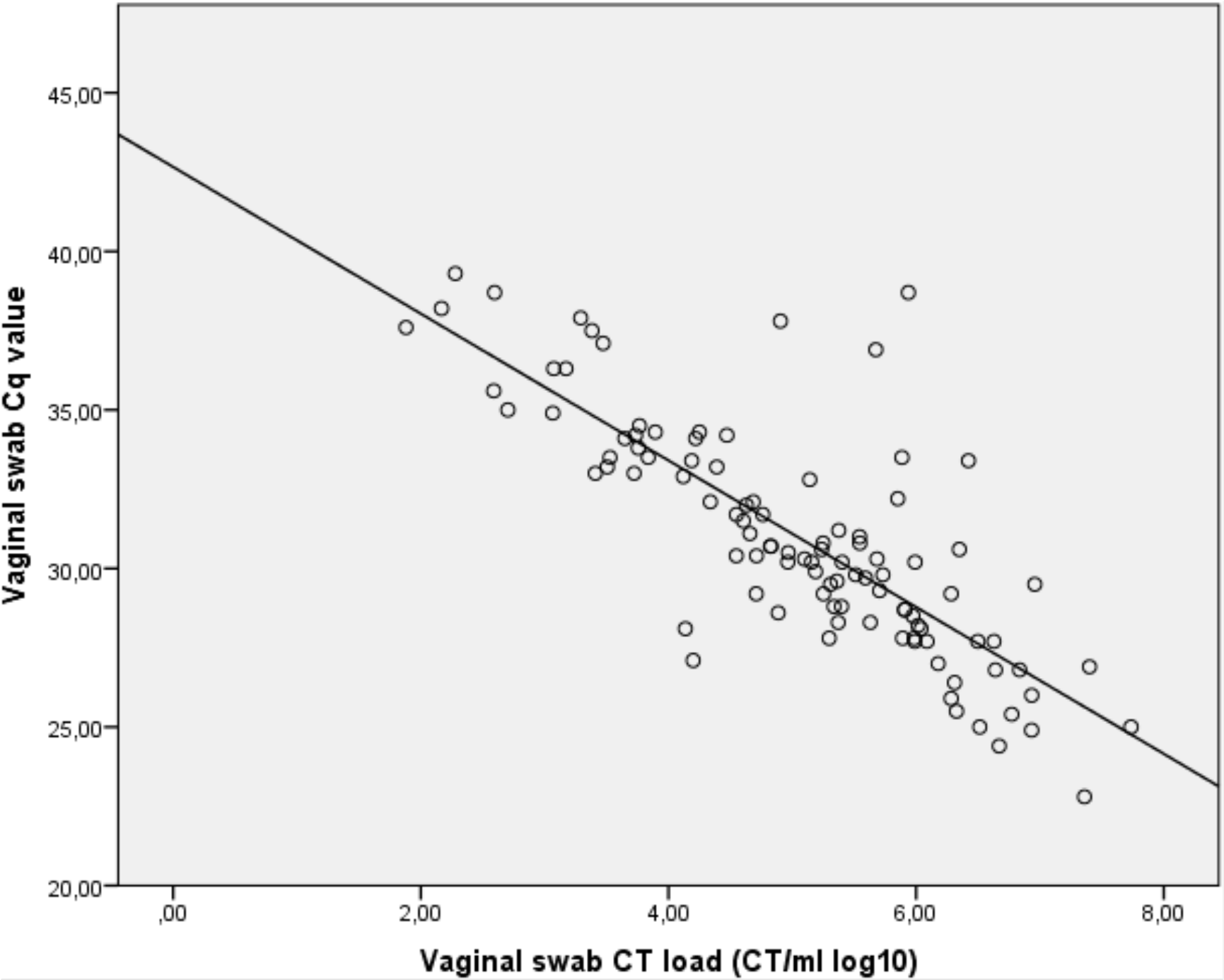

Supplement: S1 Fig — (TIF) [file pone.0215606.s002.tif]
